# Recent Trends in the Management of Bladder Exstrophy: The Gordian Knot Has Not Yet Been Cut

**DOI:** 10.3389/fped.2019.00110

**Published:** 2019-03-29

**Authors:** Martin Promm, Wolfgang H. Roesch

**Affiliations:** Department of Pediatric Urology, Clinic St. Hedwig, University Medical Center of Regensburg, Regensburg, Germany

**Keywords:** bladder exstrophy, epispadias, urologic surgical procedures, delayed closure, osteotomy

## Abstract

Although enormous effort has been made to further improve the operative techniques worldwide, the management of bladder exstrophy (BE) remains one of the most significant challenges in pediatric urology. Today it is universally agreed that successful and gentle initial bladder closure is decisive for favorable long-term outcome with regard to bladder capacity, renal function and continence. Due to a number of reasons, including a lack of comparable multicenter studies, a range of concepts is currently used to achieve successful primary closure. We review the literature of the last 15 years on the current concepts of bladder exstrophy repair with regard to the time of primary closure (initial vs. delayed closure), the concepts of primary closure (single-stage vs. staged approach; without osteotomy vs. osteotomy) and their outcomes. There is a worldwide lack of multicenter outcome studies with adequate patient numbers and precisely defined outcome parameters, based on the use of validated instruments. The modern staged repair (MRSE) in different variations, the complete primary reconstruction of exstrophy (CPRE), and the radical soft-tissue mobilization (RSTM) had been the most extensively studied and reported procedures. These major concepts are obligatory stable now for more than 20 years. Nevertheless, there are still a lot of open-ended questions e.g., on the potential for development of the bladder template, on continence, on long-term orthopedic outcome, on sexuality and fertility and on quality of life. Management of BE remains difficult and controversial. Further, clinical research should focus on multi-institutional collaborative trials to determine the optimal approach.

## Introduction

Today the diagnosis of bladder exstrophy (BE) is usually made by prenatal ultrasound screening or by inspection after birth. In classic BE the bladder is completely opened in the lower abdomen so the edge of the inner surface of the bladder is fused to the abdominal skin. The evaginated bladder template is of different individual size. The mucosa of the bladder appears reddish and polyps may be seen on it. The symphysis is widely separated. In male an epispadic urethral plate covers the whole dorsum of the penis from the bladder template to the glanular grove (1). In females, the clitoris is split and is located next to the open urethral plate. The vaginal opening appears narrow and is placed anteriorly on the perineum (1).

Often pediatricians are consulted to assess the neonates, to initiate further diagnostics and to refer them to pediatric surgeons or pediatric urologists. Due to the very low prevalence and various treatment approaches of this disorder, most physicians are not familiar with a standardized procedure.

The aim of BE repair is successful bladder closure and penile reconstruction in order to provide a capacious low-pressure and competent functioning reservoir as well as a good cosmetic appearance of the genitalia with unimpaired function and unobstructed urethra. By a successful primary closure normal renal function should be preserved.

The management of BE remains one of the greatest challenges in pediatric urology. While it is universally agreed that successful and gentle initial bladder closure is of utmost importance for development of bladder capacity and continence there are still numerous different concepts for the initial management of this condition (2). The main issues discussed are the time of primary closure (immediate vs. delayed closure), the type of BE repair (complete or staged), and finally the need of symphysis approximation with or without pelvic osteotomy.

Beyond doubt irrespective of the kind of reconstruction technique worldwide attempts are made to reduce the morbidity of management concepts.

## Timing

Regardless of the different surgical techniques, timing of primary closure still remains a matter of debate. The initial closure may be performed within the first 48–72 h of life (immediate) or at ~6–12 weeks of age (delayed).

Early closure is recommended to prevent environmental injury of the bladder mucosa (3). However, the impact of early closure in respect of the incidence of inflammation, fibrosis, or even malignant changes remains unclear. Rösch et al. characterized the histology of polyps and mucosal biopsies excised during primary delayed surgery (4) and compared their findings with previous data concerning biopsies obtained during early closure in the neonate. In comparison to the specimens of newborns with BE (5) active inflammation was more common but fibrosis and more severe inflammation was not more frequent in delayed closure. Ferrara et al. suggested that some microscopic changes, such as squamous metaplasia, reverse to normal after bladder closure (6, 7). Literature on mucosal changes in early life in BE is rare. Including data of subsequent series (8, 9), there is no advice for histologically or immunohistochemically detectable premalignant changes after the interim of 6–8 weeks and in comparison to early bladder closure neither fibrosis nor more severe inflammation seems to be more frequent after that time.

Anesthesia and analgesia are challenging in primary BE repair especially in early closure. Some factors associated with perioperative cardiac arrest have been identified (10, 11). It was found that the largest number of perioperative complications occurred in newborns (10, 12). Further, on there is a higher oxygen uptake rate in newborns. This means a severely increased risk of hypoxia damage in cases of circulation or ventilation problem during surgery or post-operatively. One of the most important determinants of successful bladder closure is effective local analgesia. There is evidence that neonates exposed to extreme stress during delivery, or to a surgical procedure, react to later noxious procedures with heightened behavioral responsiveness (13). The use of continuous caudal epidural analgesia allows application of local analgesia minimizing the use of intravenous and oral opiate analgesia (14). It also helps to wean the babies from the respirator and decreases pediatric intensive care unit length of stay furthermore the minimal use of opiates may also decrease gastrointestinal motility disturbances (2). In general neonatal epidural analgesia is feasible but it is a given fact that the application of an epidural catheter in a 6-weeks old infant is more reliable.

Also with regard to the development of renal function there is a more stabilized situation after the 6th week of life (15):

- Acid-base-regulation in the neonate is characterized by a reduced threshold for bicarbonate reabsorption. There is also an inability to respond to an acid load, this improves by 4–6 weeks postnatally.
- Renal concentration capacity is reduced in the first 2 month of live.
- In the neonate glomerular filtration rate (GFR) is low and doubles in the first 2 weeks and doubles again in the following 2–3 weeks.

This immature situation of renal function in the newborn period means a high risk for long-term kidney function. Even marginal iatrogenic fluid imbalance or temporary post-renal obstruction (e.g., stent or catheter obstruction) may provoke irreversible renal impairment.

Last but not least bonding after birth is of eminent importance of developing infant's self-regulation and further interaction to mother and father (16, 17). In particular, separation may delay and disrupt bonding in parents. Another advantage of delaying surgery is initiating breastfeeding (18, 19). In addition, the time between birth and initial repair is useful to the parents to get psychological support if desired and to prepare themselves for the procedure and the lengthy recovery period following.

## Preoperative Management

In case of delayed management, only a few diagnostic measures are required preoperatively. Besides the ultrasound of the upper urinary tracts and the hips an echocardiography is recommended, recent studies indicate that there is an increased risk of associated congenital heart failures in BE patients (20). Further diagnostics like MRI or computer tomography are not necessary. Until surgery the bladder template is covered with topical ointment compresses against inflammation and alteration of the mucosa (1). There is no need for an extended hospital stay after delivery or even stay on the intensive care unit. Antibiotic prophylaxis is not necessary and not recommended in order to avoid development of resistance or topical fungal infection.

## Main Surgical Concepts

Already at the beginning of the twentieth century there are first reasonable attempts to treat this defect surgically. Since the 50's numerous different concepts are introduced to reconstruct BE under functional and aesthetic aspects.

Three of them has been the most extensively studied and reported procedures.

The modern staged repair (MSRE) (21), the complete primary reconstruction of bladder exstrophy (CPRE) (22) and the radical soft tissue mobilization (RSTM) (23).

The traditional staged reconstruction popularized by Gearhart and Jeffs has been a standard approach for many years (1, 21). The so-called **“modern staged repair” (MSRE)** is currently advocated as a modification by John Gearhart. He made this three-stage concept popular worldwide (1, 24). The bladder template the posterior urethra and the abdominal wall are closed within the first 2 days of life and the pelvic ring is adapted. Epispadias repair follows at the age of 6–9 months. In females, genital reconstruction is mostly included in the first operative procedure. As a third step, bladder neck reconstruction and simultaneously an antireflux plasty are performed when bladder capacity reaches a minimum of 85 cc. and the child is ready for continence training (1).

An antireflux plasty is always conducted with the bladder neck reconstruction (1).

Currently multiple variations of bladder neck reconstruction within this concept are established in different parts of the world. The restriction of all the above named modifications is that they can create essentially only a kind of obstruction of the bladder neck instead of a functional continence mechanism. Moreover, obstruction is not necessary for bladder growth, quiet the contrary, initial bladder neck surgery might have negative effects on the development of a functional bladder (8).

As a sort of striking a new path *Grady and Mitc*hell introduced the **complete primary repair of bladder exstrophy (CPRE)** in hope it would more closely mimic the normal anatomy and therefore physiology of the normal bladder (22, 25). This approach includes bladder closure and reconstruction of the penis using the penile disassembly technique. This procedure is implemented on the basic concept that the primary defect of bladder exstrophy results from on anterior herniation of the bladder. It hence appears to be necessary to treat the bladder, the bladder neck and the urethra as one entity in order to transfer them successfully and permanently into the pelvis. The penile disassembly technique is performed simultaneously with bladder neck reconstruction (26). Unfortunately in the long-term follow-up in numerous cases a bladder neck reconstruction was necessary to gain social continence (27). Further on concern is raised for the risk of future detrusor underactivity as well as erectile function due to the “unimpeded radical mobilization” of the bladder-urethral plate complex in the direction of the pelvis (25).

The **radical soft tissue mobilization (RSTM)** introduced by Kelly (23) may be considered as the so far most consequent concept off the classical bladder neck reconstruction. The unique aspect of this technique is the dissection especially of the pelvis and the corpora cavernosa from the ischiopubic rami including the periosteum with the attachments of the voluntary and unvoluntary sphincter muscles and the pudendal vessels and nerves (23). These muscles are used as a wrap around the new created posterior pelvic urethra to work as a continence mechanism. No osteotomy is performed since RSTM allows sphincter reconstruction and abdominal wall closure without tension.

RSTM is an anatomical reconstruction of BE generally performed as part of a two-staged strategy following successful neonatal closure. Complete delayed bladder closure with RSTM is a recently published modification of this concept (6).

However, the Kelly repair remains a long and technically challenging procedure even in experienced hands with a very possible risk of ischemic damage of the erectile tissue (28). Further on, leaving the symphysis without adaptation poses a certain risk with regard to the long-term abdominal wall stability and the gynecological outcome, during pregnancy as well as in terms of early prolapse of uterus (29).

## Continence Results

Although there are numerous publications on BE, most of the outcome are recorded retrospectively as single-center or single-surgeon-studies. Different definitions observation periods, end-points, and successful outcome, in particular the definition of “continence” and possibly further surgeries lead to quit different results (Table 1). Although first results of all approaches show a very promising high rate of continence, long-term studies that must mean at least 20 years of follow-up (36) reveal disillusioning results. This fact also seems in our experience to be more realistic. Moreover, Woodhouse et al. postulate that more than 80% of the reconstructed children can achieve continence, but there is some evidence that in 70% this is lost with time (36).

**Table 1:** Wide range of continence rate of the different approaches depending on definition of continence and observation period.

**Approach**	**Continence rate (%)**	**Literature**
MSRE	74	Gearhart et al. (30)
	62	Gupta et al. (31)
	22	Dickson et al. (32)
CPRE	80	Grady et al. (22)
	74	Hammouda et al. (33)
	23	Arab et al. (27)
RSTM	73	Kelly et al. (23)
	70	Jarzebowski et al. (34)
	33–67 (female) 44–81 (male)	Cuckow et al. (35)

## Epispadias Repair

The following procedures are the basis to ensure a functional and cosmetically acceptable outcome (1):

- The remove of dorsal chordee
- Reconstruction of the urethra
- Glandular reconstruction
- Penile skin closure

*Silver* was able to show that the corpora cavernosa in BE are very much shorter than in age-matched controls (37). The reduced length of the penis is thus primarily an acquired deficit of corpora cavernosa tissue and not only a consequence of the chorda and the bilateral fixation to the ascending pubic rami, which was assumed for a long time.

**Figure 1:**
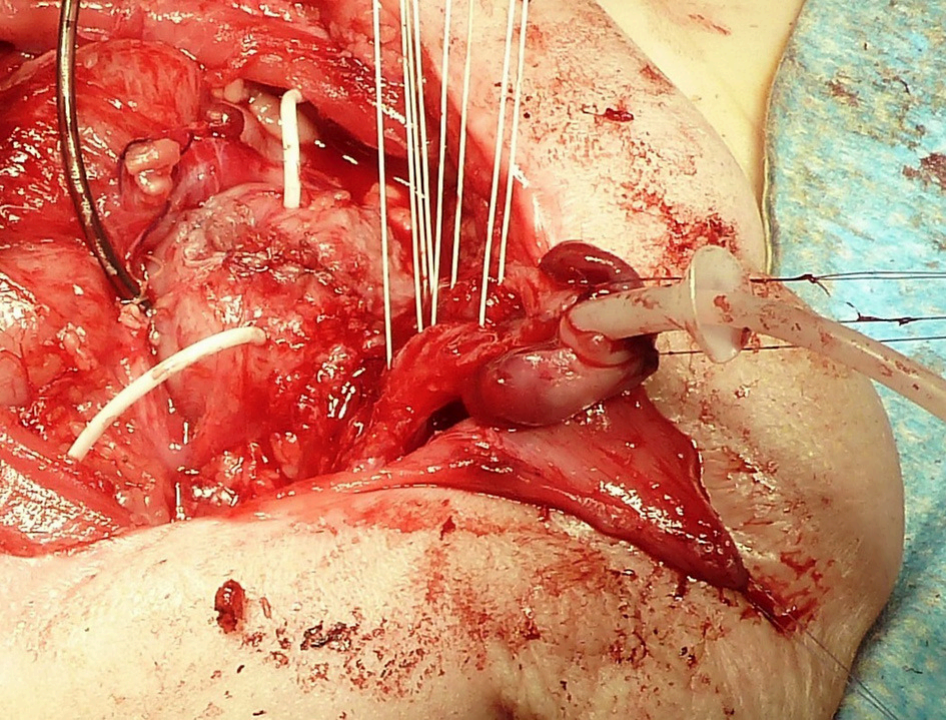
Bladder after primary closure drained by a suprapubic and two ureteral catheters. Four sutures are prepared for approximation of the separated corpora carvernosa over the tubularized urethra with an indwelling stent. The corpora are rotated laterally to correct the dorsal deviation of the penis.

*Ransley* introduced the concept of releasing dorsal chordee by incision and dorso-medial anastomosis of the corpora cavernosa above the urethra (38). Today, the *Cantwell-Ransley technique* is a modification and further development in which a very much more effective relocation of the urethra between or below the corpora is possible by complete mobilization of the urethral plate from the corpora (Figure 1).

The characteristic feature of the *Mitchell technique* is the complete dissection of the penis into 3 parts (26): The urethral plate, the right corpus cavernosum with hemiglans and the left corpus cavernosum with hemiglans (26). After tubularization of the urethral plate, it is positioned ventrally between the corpora cavernosa. If the urethra is too short, the neomeatus hast to be positioned on the ventral part of the penis. Hence, most patients require an additional procedure for hypospadias repair. Further on concern is raised for future erectile function due to shearing and stretch injury to the nerve fibers during complete penile disassembly (25).

Perineal dissection during the RSTM allows complete exposure of the corpora cavernosa (23). Incision of the periosteum of the ischio-pubic rami until the Alcock's canal allows a full mobilization of both corpora. Mostly urethral plate is short and would retract the corpora and shorten the penis. Therefore, in most cases distal urethra is disconnected from the glans and placed in hypospadic position. After readaptation the corpora were anchored to the neosymphysis using unabsorbable sutures.

Owing to extensive mobilization, all techniques of epispadias repair have in common that they require a very meticulous dissection in the anatomical layers using magnification glass in order to maintain the blood and nerve supply of the individual structures to avoid erectile dysfunction and corporal atrophy.

Nowadays, the reconstruction of **female genital** becomes less invasive. The split clitoris is usually left untouched to protect the delicate nerve supply and to avoid scaring caused by later re-dehiscence of the symphysis. Recently Benz et al. were able to show that contrary to the corpora cavernosa in boys, girls with BE have the majority of the clitoral body anterior to the pelvic attachment (39). Skin and tissue retraction in the mons pubis area is cosmetically improved by mobilizing adjacent inguinal tissue and rotating it medially into the affected area. In about 2/3 of these patients vaginoplasty is advisable (1). Episiotomy or an introitusplasty using a triangular skin-flap (*Fortunoff-flap)* can be performed to prevent repeated dilatations during childhood (1, 40). This should be done just in before or during puberty.

## Need of Osteotomies

The **role of osteotomy** is still a main topic in initial bladder closure. For a long time osteotomy was regarded essential for a successful outcome. But there are also reports confirming no difference in success of bladder closure (1, 41, 42). However, it is known that symphysis diastasis recurs after all commonly used pelvic closure techniques (43). There are only a few studies dealing with pubic diastasis after various types of pelvic osteotomy in a reasonable follow-up (36–39). According to these data the distance of recurrent mean pubic diastasis is not differing relevant in the long-term with and without osteotomy (Table 2). Castagnetti et al. compared patients after initial closure with and without osteotomy prospectively (46). In the long-term follow up they found no significant difference in the wide of pubic diastasis, in the number of exstrophy-related surgical procedures, in the incontinence rate and in the number of patients needing clean intermitting catheterization for bladder emptying (46). Kertai et al. was able to show that despite BE-specific hip morphology, long-term hip function was not impaired in adult adolescent patients after symphysial approximation without osteotomy in infancy. The symphysis diastasis after this procedure was also comparable to available post-osteotomy data in the long-term (43). In a case series published by Mushtaq et al. (2), primary bladder closure without osteotomy and post-operative immobilization was successful in 70 of 74 patients (95%) in respect to bladder closure. In our department pelvic ring closure could be achieved during the last 15 years without osteotomy in all infants with classical BE younger than 8 weeks (1, 41).

**Table 2:** Outcome of symphyseal approximation with and without osteotomy.

**Literature**	***N*=**	**Median age at investigation**	**Type of osteotomy**	**Symphysis with cm (range)**
Kaar et al. (44)	13 (11 m., 2f.)	24 years (17–36 year)	Posterior osteotomy	5.8 cm (4.1–11.2)
Satsuma et al. (45)	9 (3m., 6f.)	8 years (5 month−17.5 year)	Anterior or combined osteotomy (*n* = 3) Posterior osteotomy (*n* = 6)	3.75 cm (1–7)
Castagnetti et al. (46)	14	9.7 years (3.1–17.8 year)	No osteotomy (*n* = 6) Osteotomy	4.9 cm (2.4–6.6)
			various types (*n* = 8)	4.2 cm (2.5–10.1)
Kertai et al. (43)	17 (14 m., 3f.)	18.2 years (13–28 year)	Symphysis adaptation without osteotomy	5.1 cm (2.8–8.5)

In female patients symphysis approximation may prevent uterine prolapse regardless of the type pelvic adaptation (with or without osteotomy) (1).

Nevertheless, based on the available literature and contemporary variability in worldwide practice, it would appear that there is currently no consensus regarding the necessity of osteotomy in primary BE repair. Although not universal, most would agree on the efficacy of osteotomy in redo cases (47).

## Current Research Gaps and Potential Future Developments

A critical look into the historical data indicates that almost nothing is new in the philosophy and treatment of BE since more than one century ago. Nevertheless, due to the benefits of new technological developments there was an appreciable progress in BE reconstruction during the last decades of the last century. All these major concepts are obligatory stable now for more than 20 years and ensure a safe primary bladder closure including an appealing appearance of the genitalia in experienced hands. Apart from that, there are still a lot of open ended questions e g., on the potential for development of bladder capacity, on continence, on long-term orthopedic outcome, on sexuality and fertility and on quality of life.

First of all further clinical studies should focus on multi-center prospective trials with exactly defined outcome parameters to find an optimal management (29). In addition basic research is necessary to elucidate the morphological changes in the pattern of detrusor muscle and epithelium to establish a basis for understanding the preconditions for development of bladder-function and -capacity in BE.

Beside, further immuno-histologic studies of the bladder template, genetics will help to assess the prognosis in a realistic way. The systematic and comprehensive application of modern molecular genetic techniques in large BE cohorts has started to identify putative disease causing genes and regions in the genome for Mendelian and multifactorial BE phenotypes (48). Such studies can offer new diagnostics, and provide a more exact estimation of recurrence risk in affected families (48). Parallel functional analysis of the respective embryonic pathways offers a more profound understanding of the molecular mechanisms underlying the embryology of the urogenital tract (48). Moreover, understanding the respective embryonic pathways can help to explain related genitourinary malformations (49).

Tissue engineering aims to develop alternatives for current techniques in which intestinal tissue is used for patients with inadequate development of bladder capacity. Recent studies using tissue engineered extracellular matrices or acellular scaffolds with growth factor in animal models are promising (50, 51). However, there are scores of open issues which need to be fully clarified and defined before tissue-engineering in urology progresses from bench to bedside in BE-reconstruction.

Muscle-derived stem cells (MDSC) may offer further benefits in regenerative medicine (52). Several clinical studies have evaluated the effect of cell therapy with autologous myoblasts in the treatment of urinary incontinence, and have shown promising results (53, 54). Against this background MDSC therapy might represent a minimally-invasive procedure also in the treatment of patients with isolated epispadias in the near future. Latest studies are promising to generate differentiated urothelium from stem cells isolated from the urine. Urothelium obtained this way seems to be comparable with native urothelium and provides a valuable tool for reconstruction of the urinary tract as well as offers the chance for further studies in urothelial dysfunction (55).

Management of BE remains difficult and controversial. Further basic and clinical research should focus on multi-institutional collaborative trials to determine the optimal approach. Irrespective of that multidisciplinary ideation is in demand to generate new functional reconstruction concepts for this condition.

## Author Contributions

All authors contributed conception and design of the review, wrote sections of the manuscript, contributed to manuscript revision, read, and approved the submitted version.

### Conflict of Interest Statement

The authors declare that the research was conducted in the absence of any commercial or financial relationships that could be construed as a potential conflict of interest.
